# Time Course of Current of Injury Is Related to Acute Stability of Active-Fixation Pacing Leads in Rabbits

**DOI:** 10.1371/journal.pone.0057727

**Published:** 2013-03-05

**Authors:** Shalaimaiti Shali, Alimujiang Wushou, Entao Liu, Lin Jia, Ruiming Yao, Yangang Su, Junbo Ge

**Affiliations:** 1 Department of Cardiology, Shanghai Institute of Cardiovascular Diseases, Zhong Shan Hospital of Fudan University, Shanghai, China; 2 Department of Oral and Maxillofacial-Head and Neck Surgical Oncology, College and Hospital of Stomatology of Xi’an Jiao Tong University, Xi’an, China; 3 Department of Cardiac Rhythm Disease Management, Medtronic (Shanghai) Co., Ltd, Shanghai, China; University of Minnesota, United States of America

## Abstract

**Background:**

Magnitude of current of injury (COI) consequent to pacemaker lead fixation is recognized as a predictor of acute lead stability. It is unclear whether dynamic monitoring of COI after lead fixation provides additional information beyond a single assessment performed at the time of fixation.

**Objectives:**

This study was aimed to test the hypothesis that the time course of COI is related to acute lead stability.

**Methods and Results:**

Active fixation leads with fixed screw were anchored to either Langendorff-perfused rabbit hearts endocardially or in vivo hearts epicardially in manners of contact the helix with no rotation, half rotation and full rotation, respectively. Intracardiac electrogram (EGM) was monitored dynamically from onset to resolution of COI, and magnitudes of intrinsic R wave and COI, including ST-segment elevation, ST/R and intracardiac EGM duration (IED), were measured. A digital force gauge was applied to assess lead stability. In vitro, COI in contacted leads was significantly smaller than those in half rotated (*p*<0.05) and fully rotated leads (*p*<0.05), and presented most precipitous recovery to baseline (1.5±1.1 min, *p*<0.05). Half-rotated and fully rotated leads manifested the same magnitude of COI right after placement. However, the time course of COI was significantly longer in fully rotated leads than that in half rotated leads (26.5±2.8 min *vs.* 5.6±2.0 min, *p*<0.05). Similar findings were observed in vivo. The time course of COI was significantly correlated with the force needed to detach the lead from myocardium (*r* = 0. 72, n = 48, *p*<0.001).

**Conclusions:**

Time course of COI is related to acute lead stability in rabbits. One might be misled by a single assessment of COI magnitude right after lead placement, whereas persistence of COI is likely to be a useful indicator of adequate lead stability.

## Introduction

Adequate lead positioning is known as a key to success in pacemaker implantation, especially when selective pacing sites are preferred [1], [2]. The prevalence of lead dislodgement was reported as 1–5.2% in some observational studies [3], [4]. Permanent pacemaker leads are affixed to the myocardium either passively using tined electrode or actively by a helix. Lead implantation is inherently injurious to the focal myocardium [5]. It changes the electro-activity of myocardium similar to what ischemic injury does [6], and produces a current of injury (COI) that presented on the intracardiac electrocardium (EGM) as ST-segment elevation and increase in intracardiac EGM duration (IED) [7], [8]. Presence of COI obtained at the time of lead placement has been related to adequate performance of both passive and active-fixation leads [9], [10]. Recent studies on active-fixation leads have found that the magnitude of COI at the time of fixation is a valuable sign of desirable lead placement and satisfactory pacing threshold [11], [12]. Despite these findings, few studies so far have focused on the dynamic properties of COI after fixation. The correlation of COI time course from onset to resolution with acute lead stability still remains unclear. Therefore, the hypothesis aroused that not only the magnitude but the time course of COI may predict the adequacy of lead placement in active-fixation leads as well.

In this animal study, three patterns of active lead fixation were employed, including contacting the helix with full rotation, half rotation and no rotation. Generally, the tissue-electrode interface was more pronounced as the lead helix was given a full turn and completely embedded in myocardium than in case of half rotation, while contacting the lead without rotation only causes minor electrode pressure against the endocardium or epicardium [9]. Hence, these patterns of lead positioning employed in the present study could mimic decremental states of lead stability. We chose the rabbit in our experiments which has been widely used in cardiac electrophysiology studies. Rigorous measurements of COI were performed dynamically throughout the procedure in both in vitro and in vivo models to investigate the significance of COI time course on acute lead stability. In our experimental settings, we found a trend to decrease in COI magnitude with different timings, and the time properties were associated with acute lead stability in rabbit hearts; In addition to magnitude, COI persistence may provide evidence to identify lead stability. To our best knowledge, this is the first systematic report on time properties of COI and its potential role in predicting acute lead behavior. Of course, the results obtained are only true for rabbits, and are not transferable to humans without further research.

## Materials and Methods

This study conformed to the guiding principles for the Care and Use of Laboratory Animals published by the US National Institutes of Health (NIH Publication No. 85–23, revised 1996). All animal protocols were approved by the Institutional Animal Care and Use Committee of Zhongshan Hospital, Fudan University, PR China. Twenty four adult (2–2.5 months) male New Zealand White rabbits, weighing 3.2∼3.6 kg, were used. Sixteen of them were randomly assigned to in vitro experiment, and the remainder to in vivo study.

### Heart Preparation and Pacemaker Lead Implantation

#### In vitro

Rabbits were anesthetized with intravenous sodium pentobarbital (50 mg/kg) after intravenous heparin injection (1000U). After exposing the thorax, the heart was quickly isolated, and underwent constant Langendorff perfusion with oxygenized thermostatic Krebs-Henseleit solution heated to 37°C as previously reported [13]. The solution consisted of NaCl, KCl, NaHCO_3_, Glucose, KH_2_PO_4_, MgSO_4_·7H_2_O and CaCl_2_. The active-fixation leads with fixed helix (Medtronic3830, Minneapolis, USA) were advanced through a minor incision of 5 mm on the left ventricular (LV) lateral wall into LV cavity. The lead diameter was 1.4 mm, and the length and diameter of helix electrode were 1.8 mm and 1.03 mm, respectively. Three areas, including LV basal anterior wall, basal inferior wall and basal septum, were chosen for lead positioning. The screw in pacemaker lead was attached to myocardium with three approaches, each for one area in a random fashion: holding in contact with endocardial surface (contact), giving two complete turns in a clockwise direction (half rotation), and giving four complete clockwise rotations to fully embed the electrode into LV myocardium (full rotation). A minimum distance of 10 mm was required between adjacent sites in the same heart. In brief, LV basal anterior wall was determined first epicardially, and then the pacing lead was directed to the previously decided area when advancing into LV cavity. Once the lead was attached to the endocardium, lead location was confirmed by touching the corresponding point on epicardium to feel the tip of lead against a finger. Lead was affixed to the basal inferior wall in the same manner. Distance between two locations was determined epicardially as a rough estimation on endocardium. As for the septum, lead was directed from the incision perpendicularly to the opposite wall (the septum). Similarly, distance between two locations on the basal inferior or anterior wall and septum was estimated by the shortest epicardial length from the insertion site on basal inferior or basal anterior wall to the corresponding posterior or anterior interventricular groove. If two positions were too close, less than 10 mm, the septum location would be canceled. The pacing leads were then connected to a pacing system analyzer (Medtronic2290, Minneapolis, USA) for intracardiac EGM recording.

#### In vivo

General anesthesia was performed using intravenous sodium pentobarbital (50 mg/kg). The rabbits were intubated and mechanically ventilated using room air. The heart was exposed via left lateral thoracotomy. Model 3830 pacing leads were then respectively fixated to three regions of epicardium, including right ventricular (RV) basal anterior wall, LV basal anterior wall and the apex, in the same manners as performed in vitro. Special attentions were paid to avoid damages to coronary vessels. The subsequent intracardiac EGMs were acquired as well.

### Intracardiac EGM Measurements

Real-time Intracardiac EGM was monitored by pacing system analyzer with specific settings as follows: a bandpass filter setting of 0.5 to 250 Hz, a sampling rate of 9,862 samples per second per channel, a resolution of 0.2 mV or 10%. Continuous acquisitions of intracardiac EGM were performed up to 5 min in contacted leads, 10 min in half rotated leads and 30 min in fully rotated leads in vitro, and 10 min in contacted leads, 30 min in half rotated leads and 60 min in fully rotated leads in vivo. Three or four representative beats were recorded on the paper recorder with 1 min interval at a speed of 200 mm/s. Measurements including the amplitude of peak to peak R wave, the maximum amplitude of ST-segment elevation from baseline, and the value of IED from onset to termination of intracardiac EGM were measured manually by two individuals who were blinded to the study design, and then averaged. The magnitude of ST-segment elevation was divided by R wave amplitude and presented as ST/R. According to a prior study, ST-segment elevation more than 25% of intrinsic R wave amplitude was defined as a sign of COI [12]. Therefore, the time when ST/R dropped to 0.25 corresponded to the point of COI resolution. The time interval from COI appearance to recovery was calculated as the time course of COI.

### Acute Lead Stability Assessment

After each complete acquisition of intracardiac EGM, a digital force gauge (Model SH-10, Shan Du, China) linked with half or fully rotated leads was applied horizontally in uniform motion. The strength in order to detach the lead from myocardium was tested.

### Data Analysis

STATA 10 (Stata Corp LP, College Station, TX, USA) software was used for data analysis. Continuous variables were presented as means ± standard deviations. The sample size is estimated aiming an 80% power (two-sided alpha, 0.025) to detect a difference of 2 mV in ST-segment elevation. T test and Wilcoxon rank-sum test were used to compare the means between two groups. One-way ANOVA test and Friedman test were used to compare the means of multiple groups. The Spearman rank correlation coefficient was used to examine the presence of correlations between quantitative variables. A p value of <0.05 was considered statistically significant.

## Results

**Figure 1:**
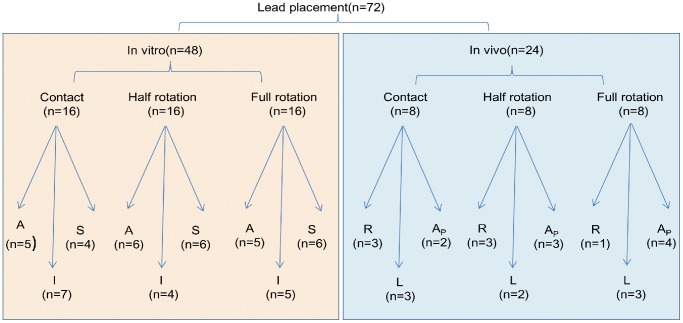
Illustration of the lead location in each group. A: anterior basal wall, I: inferior basal wall, S: septum, R: right ventricular anterior basal wall, L: left ventricular anterior basal wall, A_P_: apex.

**Figure 2:**
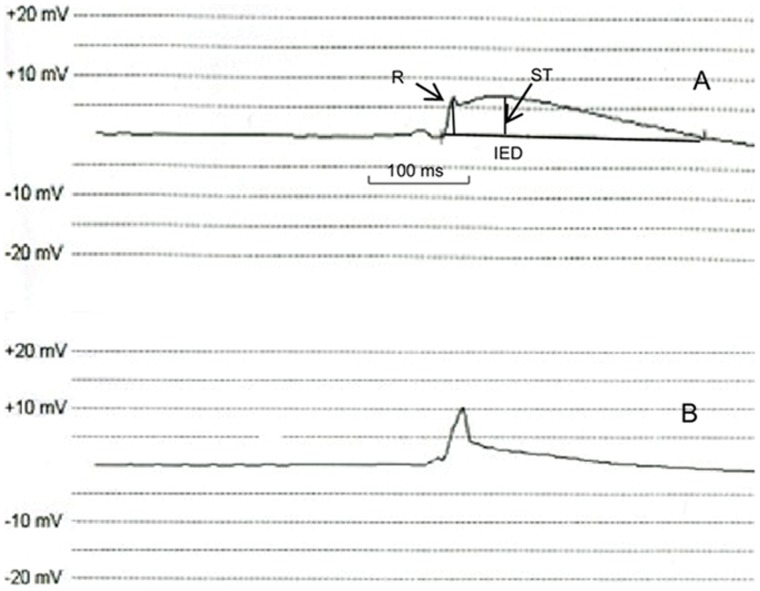
Example of intracardiac electrogram recorded by pacing system analyzer (200 **mm/s).** A. Onset of COI, manifested as ST-segment elevation after R wave deflection and increase of intracardiac electrogram duration (IED). B. Resolution of COI, ST-segment declined to less than 1/4 of R wave amplitude, and the significant decrease in IED also occurred.

Sixteen rabbit hearts underwent successful Langendorff perfusion, and eight in vitro hearts maintained normal activity during anesthesia. In total, lead positioning was performed 72 times, including 48 in vitro (n = 16 for each contacting, half rotating or fully rotating approach) and 24 in vivo (n = 8 for each approach). Details about lead locations with different approaches in different areas were illustrated in Figure 1. 70 attempts of lead placement developed a clear COI (Figure 2A), showing a trend to decline in magnitude with time (Figure 2B). Two attempts failed to develop obvious COI exclusively when the lead was held in contact. Each distance between adjacent sites was more than 10 mm (19.2±8.6 mm.), thus no lead location was discarded.

### Dynamic Behavior and Time Course of COI

**Figure 3:**
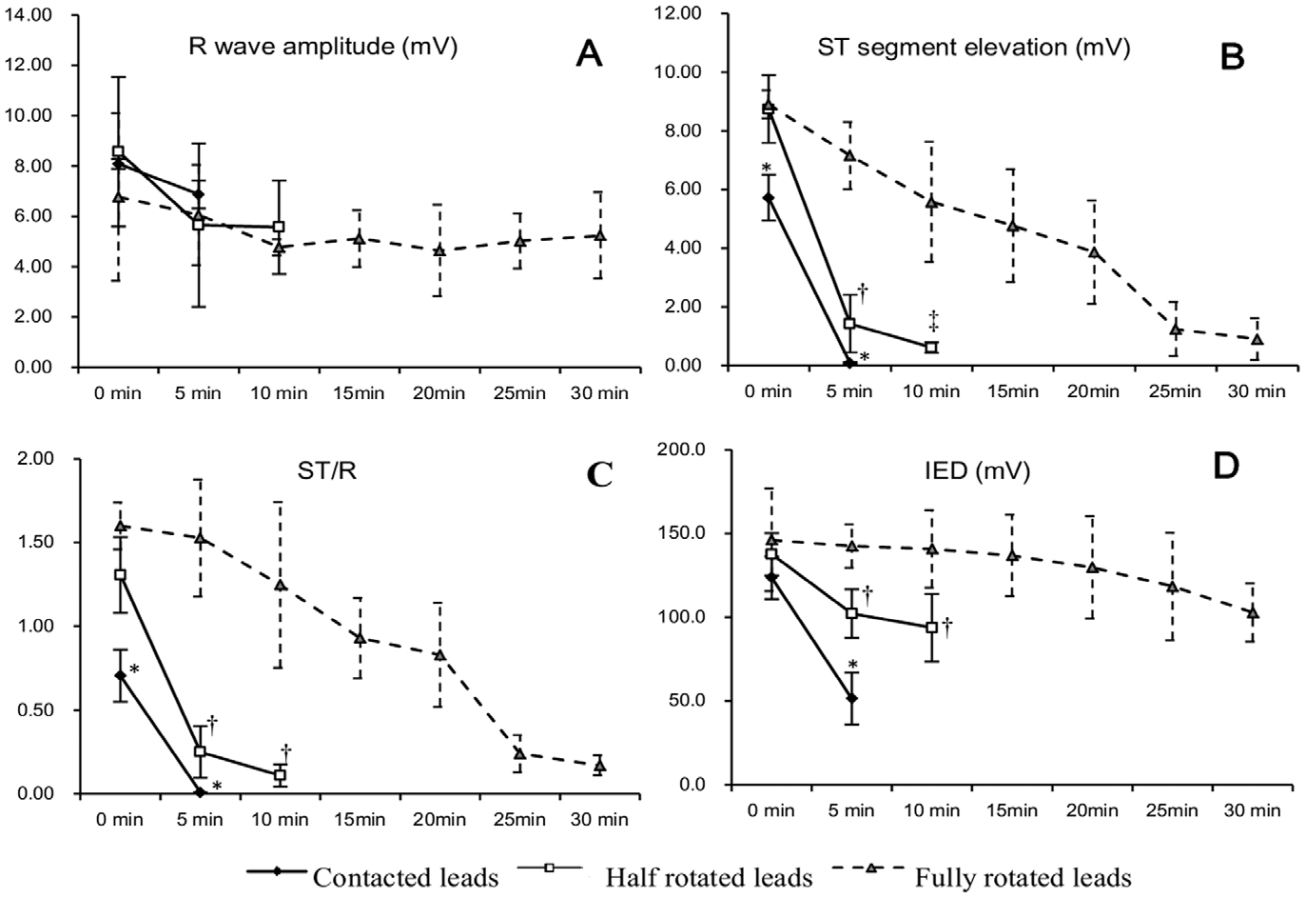
Comparison of Intracardiac EGM variables derived from isolated rabbit hearts. Panel A. Intrinsic R wave amplitude showed no significant dissimilarity regarding to different lead positionings. Panel B. Contacted leads presented the smallest magnitude of ST-segment elevation with the most rapid decline, followed by half rotated leads, while fully rotated leads showed the biggest COI amplitude and the slowest recovery. Note that there was no difference between half and fully rotated leads at 0 min. Panel C and D depicted the same findings as Panel B in the value of ST/R and IED, respectively. *: *p*<0.05 (compared with half and fully rotated leads); ^†^: *p*<0.05 and ^‡^: *p*<0.01 (compared with fully rotated leads).

In vitro, A precipitous recovery in ST-segment elevation (5.71±1.78 mV to 0.08±0.04 mV, *p*<0.01), ST/R (0.71±0.16 to 0.01±0.03, *p*<0.01) and IED (123.7±42.7 ms to 51.5±15.6 ms, *p*<0.05) were observed over up to 5 min-recording period in contacted leads (0 min *vs.* 5 min). The amplitude of ST-segment elevation in half rotated leads was 8.74±4.15 mV at 0 min and dropped to 0.61±0.18 mV at 10 min (0 min *vs.* 10 min, *p*<0.001); the value of ST/R was also reduced substantially from 1.31±0.43 to 0.11±0.07, and IED from 137.6±20.6 ms to 93.7±34.3 ms during 10 min of monitoring time (0 min *vs.* 10 min, *p*<0.05). Fully rotated leads demonstrated a slow recovery in magnitude of ST-segment elevation (8.90±2.48 mV to 0.89±0.71 mV, *p*<0.001) and ST/R (1.60±1.04 to 0.17±0.06, *p*<0.05) within 30 min-observation (0 min *vs.* 30 min), whereas the value of IED showed no significant change (Figure 3, Table S1, S2, S3). Accordingly, time course of COI was significantly longer in half rotated leads than that in contacted leads (5.6±2.0 min *vs.* 1.5±1.1 min, half rotated *vs.* contacted, *p*<0.05), and significantly in fully rotated leads compared with that in half rotated leads (26.5±2.8 min *vs.* 5.6±2.0 min, fully rotated *vs.* half rotated, *p*<0.05) (Table 1).

**Table 1:** Time course of COI from onset to resolution.

	In vitro	In vivo
Contacted leads (min)	1.5±1.1	4.1±1.2
Half rotated leads (min)	5.6±2.0*	22.3±5.8* ‡
Fully rotated leads (min)	26.5±2.8†	51.2±13.4† ‡

All data represent means± SD.

* *p*<0.05 (compared with contacted leads);

† *p*<0.05 (compared with contacted and half rotated leads).

‡ *p*<0.05 (compared with in vitro).

**Figure 4:**
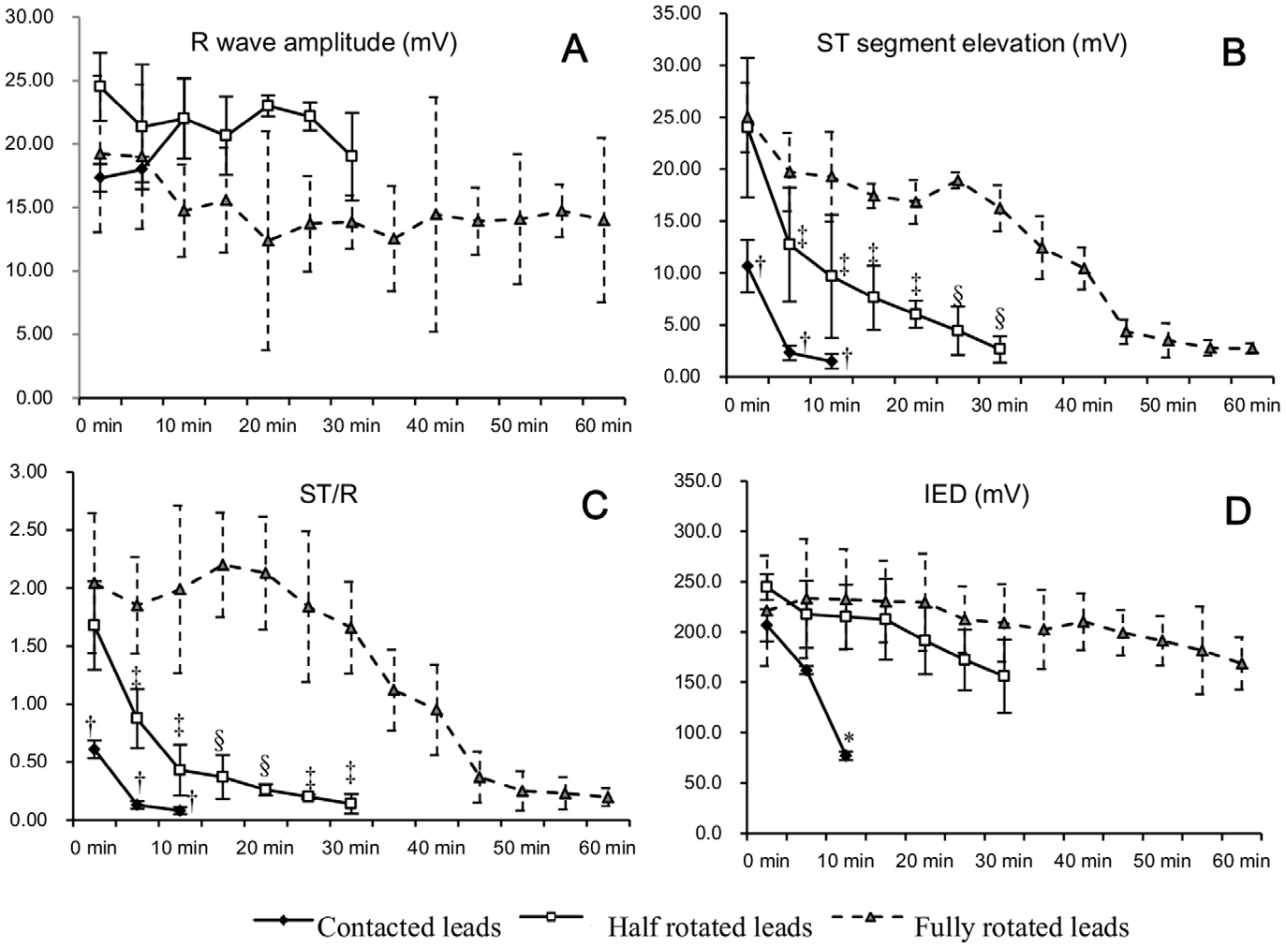
Comparison of Intracardiac EGM variables derived from in vivo hearts. Panel A, B and C showed the similar findings observed in Figure 3. Panel D. No difference in IED between half and fully rotated leads at any time point. *: *p*<0.05 and ^†^: *p*<0.01 (compared with half and fully rotated leads); ^‡^: *p*<0.05 and: §: *p*<0.01 (compared with fully rotated leads).

Similar to in vitro, all in vivo leads showed a trend toward remarkable decline in magnitude of ST-segment elevation and ST/R with different timings; however, shortening in IED during intracardiac EGM monitoring did not reach a statistical significance (Figure 4, Table S1, S2, S3). Compared to in vitro, COI in vivo lasted relatively longer in both half and fully rotated leads (in vivo *vs.* in vitro, *p*<0.05), yet the same difference failed to show statistical significance in contacted leads (Table 1).

There was no significant discrepancy in COI persistence in each group with respect to different lead locations both in vivo (basal anterior wall *vs.* basal inferior wall *vs.* basal septum, *p*>0.05) and in vitro (RV basal anterior wall *vs.* LV basal anterior wall *vs.* apex, *p*>0.05).

### Comparison of Intracardiac EGM Variables

Figure 3 graphically depicted the difference in dynamic values of COI among contacted, half rotated and fully rotated leads in vitro. In isolated rabbit hearts, R wave did not show significant difference among three approaches at any of the time points. Compared to half rotated and fully rotated leads, contacted leads produced less pronounced ST-segment elevation throughout the recording time (*p*<0.05). Although half and fully rotated leads presented similar ST-segment elevation right after fixation (8.74±4.15 mV vs. 8.90±2.48 mV, half rotated *vs.* fully rotated, *p*>0.05), their difference was significant at 5 min (3.20±1.98 mV vs. 7.16±2.15 mV, half rotated *vs.* fully rotated, *p*<0.05) and 10 min (1.29±1.18 mV vs. 5.57±2.05 mV, half rotated *vs.* fully rotated, *p*<0.01) post fixation. The value of ST/R and IED documented differences among three approaches similar to that of ST-segment elevation. Likewise, comparable findings were observed in vivo except that there was no significant difference in dynamic values of IED between half and fully rotated leads (Figure 4).

Generally, there were continuously higher amplitude of ST-segment elevation, but not ST/R, and longer IED in vivo as compared with those variables in vitro at most of the time points (Table S1, S2, S3).

Besides, Friedman test indicated a non-significant effect of different lead locations on intracardiac EGM measurements (magnitude of ST-segment elevation, ST/R and IED) in each of contacted, half rotated and fully rotated groups in vitro(*p*>0.05). The results were similar for in vivo samples (*p*>0.05).

### Correlation of COI Time Course with Acute Lead Stability

**Figure 5:**
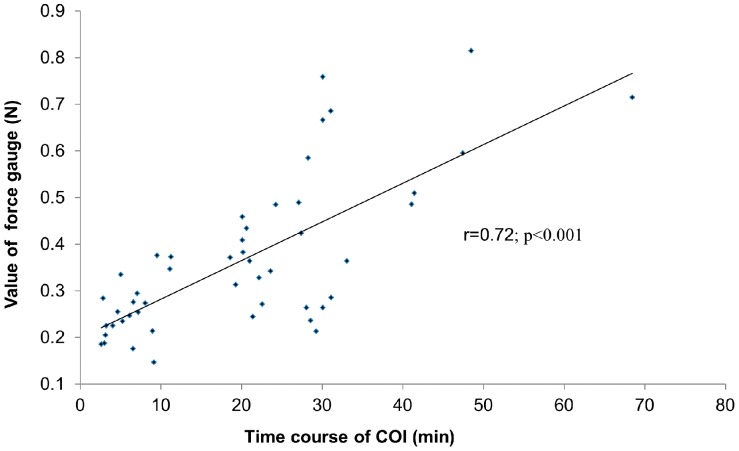
Correlation between COI time course and acute Lead Stability. COI time course from onset to resolution was significantly correlated with the force in order to detach the lead from myocardium (r = 0.72, n = 48, p<0.001).

The force applied to detach the fully rotated leads was larger than that to half rotated leads both in vitro (0.46±0.16 N vs. 0.21±0.06 N, fully rotated *vs.* half rotated, *p*<0.001) and in vivo (0.42±0.12 N vs. 0.19±0.05 N, fully rotated *vs.* half rotated, *p*<0.001). Correlation analysis revealed that there was significant positive correlation between COI time course and the stretch in order to dislodge the lead (r = 0.72, n = 48, p<0.001) (Figure 5).

## Discussion

The major finding of our study is that the time course of COI from onset to resolution is significantly correlated with acute lead stability in rabbit hearts. Dynamic monitoring of intracardiac EGM after pacing lead fixation revealed a general trend to decline in COI magnitude, but the timings were different among contacted, half rotated and fully rotated leads which reflected different levels of lead stability. Thus, persistence of COI may be a useful indicator for adequate lead stability.

Given the potential risk of extending the time of surgery, it is difficult to evaluate the overall COI time course in a clinical setting. Hence, only one study has reported the time course to COI resolution so far [11]. In this previous study, COI resolution was observed within 10 min-recording time. They found the significance of discrete COI assessed right after fixation on predicting adequate pacing threshold within 10 min, but did not further correlate the time properties with acute lead performance. In contrast, the present animal study enabled the continuous monitoring of COI for longer duration in preconditioning states of lead stability. We detected that contacted leads documented a more precipitous decline in COI magnitude than half rotated leads, while fully rotated leads were associated with the slowest COI recovery. Furthermore, there was a significantly positive correlation between the COI time course and lead stability. However, one may notice that the time lag observed in our rabbit models was longer than 10 min as previously reported [11]. Although we used the commercially available pacing lead as small as possible in diameter, it still seemed to be relatively big with respect to the size of a rabbit heart. Myocardial injury in rabbit heart was assumed to be more severe, and thus need more time for recovery than that in human heart. Therefore, this animal study was not powered to figure out the exact COI monitoring time for definite clinical guidance. Yet we believe that these findings may provide an assumption worthwhile to further investigate: relatively longer time to decrease in COI magnitude might promise better lead location; on the other hand, one might consider repositioning the lead if COI presents a quick recovery.

A more recent study examined COI during the process of helix rotation that engaged Model 3830 leads into myocardium [12]. It was found that continuous monitoring of COI during fixation was of limited benefit, whereas the magnitude of COI measured at the end of fixation was strongly associated with acute lead performance. Dynamic assessment of COI after lead fixation, not during, was performed in the present study. In agreement, it was confirmed that initial measurements of COI in contacted leads were lower than those in half rotated and fully rotated leads and needed no force to remove from myocardium. However, we also noticed that the half and fully rotated leads reflecting incremental scales of lead stability did not appear to show any dissimilarity in COI magnitude at the time of fixation. Interestingly, the significant difference between them began to be clear with time. As a result, COI in fully rotated leads manifested slower recovery to baseline than that in half rotated leads. This poses an important question whether a single assessment of COI at the time of fixation may lead to overestimation of lead stability. According to our findings, the leads with a pronounced COI right after fixation may present a quick decline and be associated with poor stability; in another words, continuous monitoring of dynamic COI behavior post lead fixation was likely to confer additional benefit beyond single assessment at the time of fixation in distinguishing well-fixed leads from poor-fixed ones. However, as mentioned earlier, the proper monitoring time is unclear until further studies are conducted in clinical settings with a view to confirming these observations and validating their clinical application.

In this study, the intracardiac EGM parameters both in vitro and in vivo were examined. Since pacing leads are implanted endocardially in most of real cases, we performed in vitro study by attaching the lead to the endocardium of isolated heart. Besides, open chest observation was also conducted as in vivo hearts preserved better electromechanical function and homeostasis compared to in vitro models due to the normal blood supply instead of Langendorff perfusion. Not surprisingly, time course of COI for in vivo hearts was longer than that for in vitro hearts; values of ST-segment elevation and IED in vivo were bigger than those in vitro, and so was R wave amplitude, presumably because in vivo hearts yield stronger intrinsic signal and consequently greater COI that required longer time to resolve [14]. Nevertheless, once the value of ST/R was under consideration, which eliminated the confounding effect of intrinsic QRS signal, no difference was observed between in vivo and in vitro hearts, supporting that the value of ST/R is a useful indicator of lead performance as well. Although ST/R showed a stronger correlation coefficient than ST-segment elevation in correlation analysis, superiority of ST/R beyond ST-segment elevation is still unclear according to current evidence, and the definite predictive value of each variable remains a subject of ongoing clinical researches. Of note, despite these differences in magnitude and time properties of COI, both in vivo and in vitro observations demonstrated the same qualitative agreement between increase in COI time course and adequate acute lead stability.

The present study has a number of limitations. Given a relatively small sample size, numerous COI parameters were obtained continuously from leads positioned with different approaches at multiple sites. Therefore, positions designed for lead implanting were inconsistent with real clinical practice. In order to minimize the potential influence of multiple insertions on major findings, a minimum of 10 mm-distance between two adjacent sites was assumed according to a previous study [15]. Furthermore, distance was measured on the epicardial surface rather than endocardium which may carry a risk for metering error. Although no significant difference in measurements was found with respect to different lead locations using the same positioning approach when two insertion sites were spaced more than 10 mm apart, the accuracy of this assumption and measuring method should be further confirmed either pathohistologically or electrophysiologically. In addition, identifying lead stability by means of force gauge provides more concrete measures than the traction applied on the lead body [12]. Yet, the force gauge measurement as applied in the present study has not been prospectively validated as a precise measure of lead stability. Besides, this study did not correlate COI persistence with pacing threshold which is clinically crucial as well. Most importantly, since animal model differs markedly from the clinical situation, one should bear in mind when interpreting the results that it is still unclear whether these findings would be valid clinically. Despite these limitations above, it definitely provides hypothesis-generating insights for further clinical studies with standard implantation techniques.

### Conclusions

In conclusion, acute lead stability of active-fixation leads was correlated with the time course of COI from onset to resolution in rabbit hearts. This animal study implied that leads with a discrete COI right after fixation may present a quick decline and be associated with poor lead stability; one might be misled by the initial COI measurement right after lead placement, whereas continuous monitoring of dynamic COI behavior post fixation was likely to confer additional benefit in guiding pacemaker lead fixation. The value of COI persistence and the proper time for COI monitoring after lead placement merit further prospective investigations in a real clinical setting.

## Supporting Information

Table S1 **Intracardiac EGM variables of contacted leads in rabbit hearts.** All data represent means± SD. R: R wave amplitude, ST: ST segment elevation, IED: intracardiac EGM duration, –: data is not available. *stands for P<0.05, †indicates P<0.01 and ‡denotes P<0.001, in vivo vs. in vitro.
(DOC)

Table S2 **Intracardiac EGM variables of half rotated leads in rabbit hearts.** All data represent means± SD. R: R wave amplitude, ST: ST segment elevation, IED: intracardiac EGM duration, –: data is not available. *stands for P<0.05, †indicates P<0.01 and ‡denotes P<0.001, in vivo vs. in vitro.
(DOCX)

Table S3 **Intracardiac EGM variables of fully rotated leads in rabbit hearts.** All data represent means± SD. R: R wave amplitude, ST: ST segment elevation, IED: intracardiac EGM duration, –: data is not available. *stands for P<0.05, †indicates P<0.01 and ‡denotes P<0.001, in vivo vs. in vitro.
(DOCX)
