# Supplementary material for: Time Course of Current of Injury Is Related to Acute Stability of Active-Fixation Pacing Leads in Rabbits
Source: PLoS One. 2013 Mar 5;8(3):e57727. doi: 10.1371/journal.pone.0057727 (PMC3589396; doi:10.1371/journal.pone.0057727)
Supplement: Table S1 — Intracardiac EGM variables of contacted leads in rabbit hearts. All data represent means± SD. R: R wave amplitude, ST: ST segment elevation, IED: intracardiac EGM duration, –: data is not available. *stands for P<0.05, †indicates P<0.01 and ‡denotes P<0.001, in vivo vs. in vitro. (DOC) [file pone.0057727.s001.doc]

Table S1. Intracardiac EGM variables of contacted leads in rabbit hearts

|  | In vitro (n=14) | | | | In vivo (n=8) | | | |
| --- | --- | --- | --- | --- | --- | --- | --- | --- |
|  | R (mV) | ST (mV) | ST/R | IED (ms) | R (mV) | ST (mV) | ST/R | IED (ms) |
| 0 min | 8.09±2.00 | 5.71±1.78 | 0.71±0.16 | 123.6± 42.7 | 17.33±2.08‡ | 10.67±2.52† | 0.61±0.08 | 206.7±16.1† |
| 1 min | 7.70±3.34 | 5.19±2.36 | 0.76±0.39 | 126.6±43.0 | 20.50±5.63‡ | 8.83±1.61* | 0.44±0.09 | 211.7±14.4† |
| 2 min | 7.09±2.40 | 1.13±1.68 | 0.16±0.09 | 101.1±45.6 | 17.67±2.89† | 7.00±1.73* | 0.39±0.03 | 183.3±25.1 † |
| 5 min | 6.88 ±1.55 | 0.08±0.04 | 0.01±0.03 | 51.5±15.6 | 18.00±1.00‡ | 2.34±2.08 | 0.13±0.07 | 162.0±59.1 |
| 10 min | --- | --- | --- | --- | 22.00±3.16 | 1.50±0.71 | 0.08±0.03 | 77.0±4.2 |
| P value | NS | <0.01 | <0.01 | <0.05 | NS | <0.05 | <0.05 | NS |

All data represent means± SD.R: R wave amplitude, ST: ST segment elevation, IED: intracardiac EGM duration, ---: data is not available. *stands for P<0.05, †indicates P<0.01 and ‡denotes P<0.001, in vivo vs. in vitro.
