# Supplementary material for: Time Course of Current of Injury Is Related to Acute Stability of Active-Fixation Pacing Leads in Rabbits
Source: PLoS One. 2013 Mar 5;8(3):e57727. doi: 10.1371/journal.pone.0057727 (PMC3589396; doi:10.1371/journal.pone.0057727)
Supplement: Table S2 — Intracardiac EGM variables of half rotated leads in rabbit hearts. All data represent means± SD. R: R wave amplitude, ST: ST segment elevation, IED: intracardiac EGM duration, –: data is not available. *stands for P<0.05, †indicates P<0.01 and ‡denotes P<0.001, in vivo vs. in vitro. (DOCX) [file pone.0057727.s002.docx]

Table S2. Intracardiac EGM variables of half rotated leads in rabbit hearts

|  | In vitro (n=16) | | | | In vivo (n=8) | | | |
| --- | --- | --- | --- | --- | --- | --- | --- | --- |
|  | R (mV) | ST (mV) | ST/R | IED (ms) | R (mV) | ST (mV) | ST/R | IED (ms) |
| 0 min | 8.57 ±1.77 | 8.74 ±4.15 | 1.31±0.43 | 137.6±20.7 | 24.50±5.07† | 24.00±9.70 † | 1.68±0.38 | 244.5(12.8) † |
| 1 min | 7.78 ±2.46 | 7.33±2.70 | 1.07±0.71 | 126.7±18.0 | 24.80±2.26† | 18.65±10.08 | 0.87±0.39 | 196.5(55.4) * |
| 2 min | 6.49 ±2.16 | 5.18±0.84 | 0.90±0.48 | 115.6±18.7 | 23.65±3.94 ‡ | 16.00±8.49 | 0.69±0.36 | 205.2(53.3) † |
| 5 min | 5.65±2.85 | 1.42±0.98 | 0.25±0.16 | 102.2±24.4 | 21.35±4.92 ‡ | 12.75±7.50 * | 0.57±0.25 | 217.5(33.0) ‡ |
| 10 min | 5.57±2.06 | 0.61±0.18 | 0.11±0.07 | 93.7±34.3 | 22.00±3.16 | 9.70±5.93† | 0.43±0.22 | 215.0(31.9) # |
| 20 min | --- | --- | --- | --- | 23.00±0.82 | 6.03±1.30 | 0.26±0.05 | 191.3(33.3) |
| 30 min | --- | --- | --- | --- | 19.00±3.46 | 2.66±1.26 | 0.14±0.08 | 156.0(36.5) |
| P value | NS | <0.001 | <0.05 | <0.05 | NS | <0.05 | <0.05 | NS |

All data represent means± SD.R: R wave amplitude, ST: ST segment elevation, IED: intracardiac EGM duration, ---: data is not available. *stands for P<0.05, †indicates P<0.01 and ‡denotes P<0.001, in vivo vs. in vitro.
