# Supplementary material for: Time Course of Current of Injury Is Related to Acute Stability of Active-Fixation Pacing Leads in Rabbits
Source: PLoS One. 2013 Mar 5;8(3):e57727. doi: 10.1371/journal.pone.0057727 (PMC3589396; doi:10.1371/journal.pone.0057727)
Supplement: Table S3 — Intracardiac EGM variables of fully rotated leads in rabbit hearts. All data represent means± SD. R: R wave amplitude, ST: ST segment elevation, IED: intracardiac EGM duration, –: data is not available. *stands for P<0.05, †indicates P<0.01 and ‡denotes P<0.001, in vivo vs. in vitro. (DOCX) [file pone.0057727.s003.docx]

Table S3. Intracardiac EGM variables of fully rotated leads in rabbit hearts

|  | In vitro (n=16) | | | | In vivo (n=8) | | | |
| --- | --- | --- | --- | --- | --- | --- | --- | --- |
|  | R (mV) | ST (mV) | ST/R | IED (ms) | R (mV) | ST (mV) | ST/R | IED (ms) |
| 0 min | 6.77±2.34 | 8.90 ±2.48 | 1.60±1.04 | 146.1±30.5 | 19.23±13.16 * | 25.00±9.35 † | 2.04±2.04 | 221.2±44.8 † |
| 5 min | 6.06± 1.99 | 7.16±2.15 | 1.53±0.35 | 142.4±33.0 | 19.00±10.68 * | 19.75±3.77 ‡ | 1.85±0.15 | 196.5±59.2 ‡ |
| 10 min | 4.77±2.33 | 5.57±2.05 | 1.25±1.59 | 140.7 ±43.2 | 14.75±9.64 * | 19.28±4.32 ‡ | 1.99±1.72 | 232.3±49.6 † |
| 20 min | 4.64±1.82 | 3.86±1.77 | 0.83±0.31 | 129.7 ±30.6 | 12.38±8.62 * | 16.85±2.15† | 2.13±0.49 | 229.5±48.4 ‡ |
| 30 min | 5.24±1.72 | 0.89±0.71 | 0.17±0.06 | 102.9 ±31.4 | 13.85±9.10 | 16.25±2.11 | 1.66±1.80 | 209.0±38.5 ‡ |
| 40 min | --- | --- | --- | --- | 14.45±8.22 | 10.45±2.26 | 0.95±0.39 | 210.3±28.3 |
| 60 min | --- | --- | --- | --- | 14.00±6.48 | 2.75±0.50 | 0.20±0.08 | 168.8±28.9 |
| P value | NS | <0.001 | <0.05 | NS | NS | <0.005 | <0.05 | NS |

All data represent means± SD.R: R wave amplitude, ST: ST segment elevation, IED: intracardiac EGM duration, ---: data is not available. *stands for P<0.05, †indicates P<0.01 and ‡denotes P<0.001, in vivo vs. in vitro.
